# The Role of Pentacyclic Triterpenoids in Non-Small Cell Lung Cancer: The Mechanisms of Action and Therapeutic Potential

**DOI:** 10.3390/pharmaceutics17010022

**Published:** 2024-12-26

**Authors:** Young-Shin Lee, Ryuk Jun Kwon, Hye Sun Lee, Jae Heun Chung, Yun Seong Kim, Han-Sol Jeong, Su-Jung Park, Seung Yeon Lee, Taehwa Kim, Seong Hoon Yoon

**Affiliations:** 1 Family Medicine Clinic and Research Institute for Convergence of Biomedical Science and Technology, Pusan National University Yangsan Hospital, Yangsan 50612, Republic of Korea; dudtls0701@naver.com (Y.-S.L.); brain6@hanmail.net (R.J.K.); atgtaa08@gmail.com (H.S.L.); 2 Division of Human Biology, Fred Hutchinson Cancer Center, Seattle, WA 98109, USA; jhchung7942@gmail.com; 3 Division of Pulmonology, Department of Internal Medicine, Pusan National University Yangsan Hospital, Pusan National University School of Medicine, Yangsan 50612, Republic of Korea; yskim@pusan.ac.kr; 4 School of Korean Medicine, Pusan National University, Yangsan 50612, Republic of Korea; jhsol33@pusan.ac.kr (H.-S.J.); sujupark@pusan.ac.kr (S.-J.P.); dltmddus748@pusan.ac.kr (S.Y.L.); 5 Division of Respiratory and Critical Care Medicine, Department of Internal Medicine, Pusan National University Hospital, Busan 49241, Republic of Korea; taehwagongju@naver.com

**Keywords:** non-small cell lung cancer, pentacyclic triterpenoids, anticancer activity, chemoresistance, molecular mechanisms

## Abstract

Lung cancer remains a major global health problem because of its high cancer-related mortality rate despite advances in therapeutic approaches. Non-small cell lung cancer (NSCLC), a major subtype of lung cancer, is more amenable to surgical intervention in its early stages. However, the prognosis for advanced NSCLC remains poor, owing to limited treatment options. This underscores the growing need for novel therapeutic strategies to complement existing treatments and improve patient outcomes. In recent years, pentacyclic triterpenoids, a group of natural compounds, have emerged as promising candidates for cancer therapy due to their anticancer properties. Pentacyclic triterpenoids, such as lupeol, betulinic acid, betulin, oleanolic acid, ursolic acid, glycyrrhetinic acid, glycyrrhizin, and asiatic acid, have demonstrated the ability to inhibit cell proliferation and angiogenesis, induce apoptosis, suppress metastasis, and modulate inflammatory and immune pathways in NSCLC cell line models. These compounds exert their effects by modulating important signaling pathways such as NF-κB, PI3K/Akt, and MAPK. Furthermore, advances in drug delivery technologies such as nanocarriers and targeted delivery systems have improved the bioavailability and therapeutic efficacy of triterpenoids. However, despite promising preclinical data, rigorous clinical trials are needed to verify their safety and efficacy. This review explores the role of triterpenoids in NSCLC and therapeutic potential in preclinical models, focusing on their molecular mechanisms of action.

## 1. Introduction

Lung cancer is a leading cause of cancer-related deaths worldwide, accounting for approximately 18.7% of all cancer incidence [1]. According to the World Health Organization (WHO), more than 2.4 million new cases of lung cancer were diagnosed in 2022, and approximately 1.8 million deaths were reported [2]. The disease is primarily classified into two major subtypes: small cell lung cancer (SCLC) and non-small cell lung cancer (NSCLC). SCLC accounts for about 15% of all lung cancer cases and is characterized by its aggressive nature, rapid growth, and early metastasis [3,4,5]. It is closely related to smoking and initially responds to chemotherapy and radiotherapy but often has poor long-term survival rates due to drug resistance and relapse [6]. Meanwhile, NSCLC comprises approximately 85% of lung cancer cases and includes subtypes such as adenocarcinoma, squamous cell carcinoma, and large cell carcinoma [7]. Although NSCLC tends to grow more slowly than SCLC and is more amenable to surgical intervention in the early stages, advanced NSCLC is associated with high mortality rates due to limited therapeutic options [8]. Thus, this review focuses on NSCLC because it constitutes the majority of lung cancer cases and presents significant clinical challenges, including high mortality and a lack of effective treatments for advanced stages.

NSCLC progression involves a complex interplay of genetic and epigenetic alterations that drive tumor growth, immune evasion, and metastasis. Key pathways frequently dysregulated in NSCLC include the epidermal growth factor receptor (EGFR) signaling pathway, which promotes cellular proliferation and survival [9], and the KRAS pathway, which is linked to resistance to targeted therapies [10]. Additionally, alterations in the tumor suppressor gene TP53, present in over 50% of NSCLC, disrupt apoptosis and DNA repair mechanisms [11,12]. Other notable pathways, such as the PI3K/AKT/mTOR axis, contribute to metabolic reprogramming and angiogenesis [13,14], while the dysregulation of the JAK/STAT pathway facilitates immune evasion and chronic inflammation [15,16]. Understanding these molecular mechanisms not only provides insights into NSCLC pathophysiology but also highlights potential targets for therapeutic intervention. Despite advancements in understanding molecular mechanisms and in the development of surgical resection, radiotherapy, and chemotherapy for NSCLC [7,17,18,19], resistance mechanisms and adverse side effects continue to limit their efficacy [20,21]. While targeted therapies and immunotherapies have shown great promise [22,23], their clinical application is also hindered by challenges such as acquired resistance, insufficient biomarkers for patient selection, and substantial treatment costs [6,24,25]. Thus, there is an urgent need to identify novel therapeutic agents with enhanced specificity, reduced toxicity, and the ability to overcome resistance.

Natural products, particularly plant-derived compounds, have long been a valuable source of therapeutic agents. Recent research has highlighted the anticancer potential of pentacyclic triterpenoids [26,27,28]. These compounds, with diverse biologically active properties, have shown promise in preclinical and clinical studies [29,30]. Notably, in NSCLC treatment, these compounds exhibit diverse mechanisms of action that can complement or enhance existing treatments.

This review aims to provide a comprehensive overview of the anticancer effects of pentacyclic triterpenoids in NSCLC. It focuses on their molecular mechanisms of action, including apoptosis induction, cell proliferation inhibition, angiogenesis blockade, metastasis suppression, and the modulation of inflammatory pathways and immune responses. Examining recent studies on NSCLC highlights the therapeutic potential of pentacyclic triterpenoids and their possible use in combination with conventional therapies to overcome resistance and improve patient outcomes.

## 2. Pentacyclic Triterpenoids

**Figure 1:**
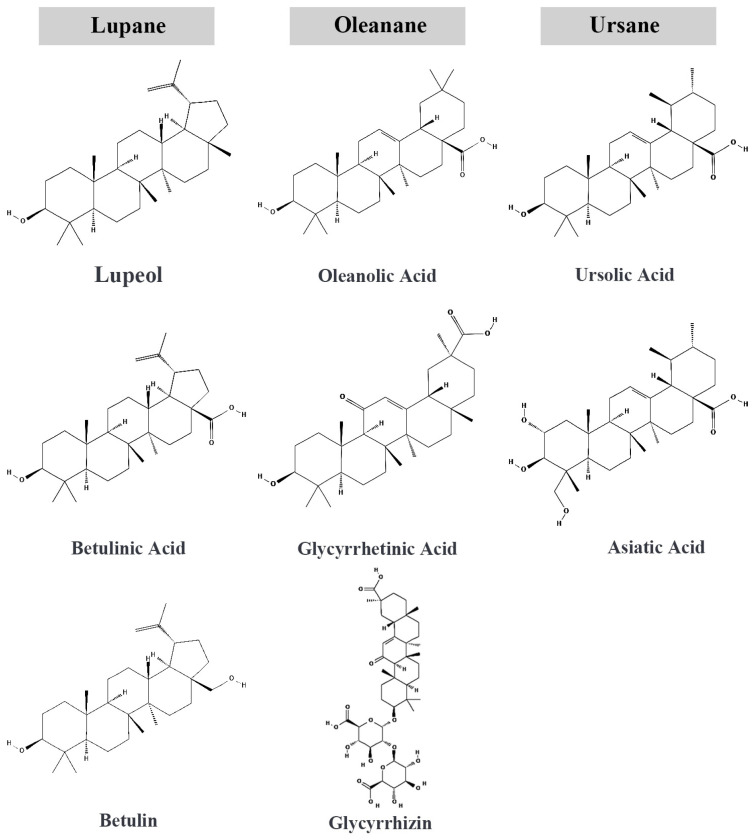
Structure of pentacyclic triterpenoids.

Triterpenoids are a large and diverse class of natural compounds derived from the isoprene unit. This unit consists of 30 carbon atoms and forms various structural frameworks, including free triterpenes, such as lupeol, oleanolic acid, ursolic acid, and squalene, and their derivatives, such as saponins and glycosides [31,32]. These compounds are widely distributed in plants, fungi, and some marine organisms, where they play protective roles against environmental stressors such as pathogens and herbivores [33,34]. Chemically, free triterpenoids exist in both cyclic and acyclic forms, with cyclic triterpenoids being particularly significant due to their stable ring structures, metabolic resilience, and broad spectrum of bioactivities [34,35]. Among these, the pentacyclic structure is the most common and is primarily found in the bark, leaves, roots, and fruits of various plants [36]. Their structure consists of five fused carbon rings with attached functional groups, enabling a wide range of biological activities such as anticancer, anti-inflammatory, and antioxidant effects [37]. Cyclic triterpenoids are further classified into monocyclic, bicyclic, tricyclic, tetracyclic, and pentacyclic forms, with pentacyclic triterpenoids being particularly prominent due to their strong anticancer properties and superior drug development potential [34]. These compounds effectively interact with cell membranes, enhancing their bioactivity, and demonstrate superior multi-target potential and metabolic stability compared to other forms of triterpenoids, making them valuable in cancer research [38]. Pentacyclic triterpenoids include lupane, oleanane, ursane, taraxerane, friedelane, and hopane types [34]. While taraxerane and friedelane exhibit anti-inflammatory and hepatoprotective effects, their anticancer potential remains less explored. In contrast, lupane (lupeol, betulinic acid, betulin), oleanane (oleanolic acid, glycyrrhetinic acid, glycyrrhizin), and ursane (ursolic acid, asiatic acid) triterpenoids have been extensively studied for their mechanisms in NSCLC, including apoptosis induction, anti-inflammatory activity, and metastasis inhibition (Figure 1). Their ease of structural modification, low toxicity, and potential for synergistic use with existing therapies make them promising candidates for focused research in NSCLC.

In addition to their well-known anti-inflammatory, antioxidant, and antimicrobial properties, pentacyclic triterpenoids have gained considerable attention for their anticancer activities [28,39,40,41,42]. The anticancer mechanisms of pentacyclic triterpenoids are diverse [26,37,43]. These compounds have been shown to inhibit various stages of cancer progression, including cell proliferation, angiogenesis, metastasis, and immune evasion, which are key hallmarks of cancer. They include the induction of apoptosis, the regulation of cell cycle progression, the inhibition of angiogenesis, and the modulation of inflammatory pathways and the immune system. The ability to target multiple molecular pathways simultaneously might make pentacyclic triterpenoids promising candidates for combination therapy and adjuvant therapy in existing treatment strategies [26,44,45,46,47].

Among these compounds, betulin and betulinic acid are derived from the lupane skeleton, but they exhibit distinct biological properties due to their structural differences. Betulin contains hydroxyl groups at C-3 and C-28, which contribute to its moderate biological activities, including anti-inflammatory and antiviral effects. On the other hand, betulinic acid, which is formed by the oxidation of the C-28 hydroxyl group to carboxylic acid, exhibits enhanced anticancer properties by efficiently inducing apoptosis in cancer cells [48,49,50,51]. Similarly, glycyrrhetinic acid and glycyrrhizin, which are derived from licorice, differ in structure and biological activity. Glycyrrhetinic acid, which is in the form of an aglycone, contains hydroxyl and carboxyl groups, which enhance its anti-inflammatory and anticancer effects. Glycyrrhizin, a glycoside of glycyrrhetinic acid, is conjugated to a glucuronic acid moiety, which improves its water solubility and systemic bioavailability, making it suitable for systemic applications [52,53,54,55,56]. The structural modifications of these compounds highlight the importance of functional groups in modulating biological activities.

Furthermore, pentacyclic triterpenoids are generally regarded as safe compounds with low toxicity profiles [57,58], which is a crucial advantage in cancer therapy. However, their limited ability to cross cellular membranes and bioavailability remain challenges for their clinical application. These limitations have led to ongoing research to improve their pharmacokinetic properties through formulation strategies and analog development [59]. In vivo studies have highlighted the promising anticancer potential of pentacyclic triterpenoids in NSCLC models. These studies have demonstrated not only significant tumor growth inhibition but also metastasis suppression, highlighting their capacity to address multiple aspects of cancer progression in preclinical animal models [60,61]. These effects are often accompanied by enhanced immune responses and reduced inflammatory markers within the tumor microenvironment [62]. These findings emphasized the potential of pentacyclic triterpenoids as effective therapeutics for NSCLC. The detailed mechanisms underlying these effects will be discussed in subsequent sections of this review.

## 3. Molecular Mechanisms of Pentacyclic Triterpenoids in NSCLC

**Figure 2:**
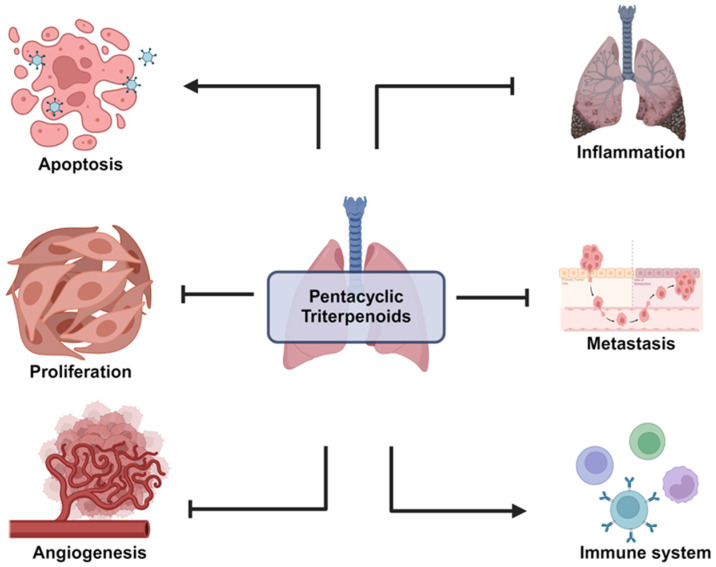
Overview of molecular mechanism of pentacyclic triterpenoids in NSCLC.

Pentacyclic triterpenoids have garnered significant attention for their anticancer properties, particularly in the context of NSCKC (Figure 2). These naturally occurring compounds, including lupeol, betulinic acid, betulin, oleanolic acid, ursolic acid, glycyrrhetinic acid, glycyrrhizin, and asiatic acid, have demonstrated various mechanisms of action that can inhibit the growth and progression of NSCLC (Table 1). The following sections explore how these compounds exert their effects through distinct biological processes.

### 3.1. Induction of Apoptosis

**Figure 3:**
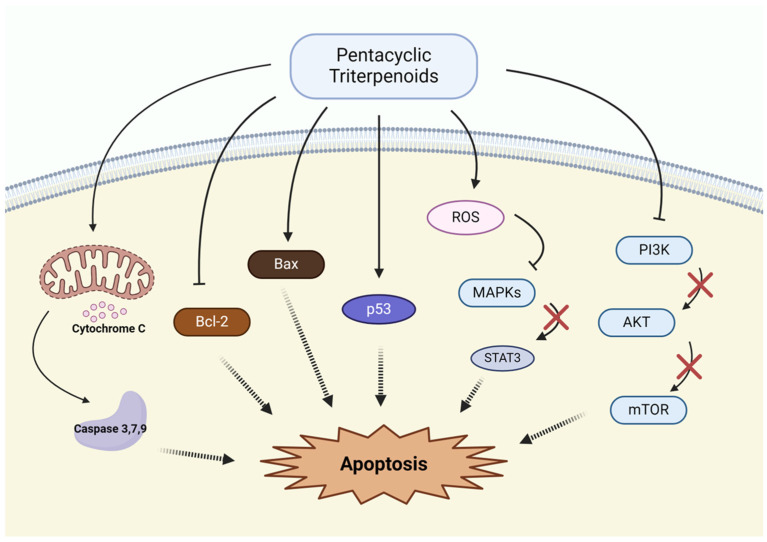
Apoptosis induction by pentacyclic triterpenoids.

Pentacyclic triterpenoids have demonstrated strong potential in inducing apoptosis, a key mechanism for eliminating cancer cells and inhibiting tumor progression. Several compounds in this class promote apoptosis by targeting both intrinsic and extrinsic pathways, disrupting mitochondrial integrity, and modulating apoptosis-regulating proteins (Figure 3).

Lupeol, a compound found in various plants such as mangoes and olive oil, induces apoptosis via both mitochondrial and extrinsic pathways by upregulating pro-apoptotic proteins such as Bax and downregulating anti-apoptotic proteins like Bcl-2 [63,64,65,66]. Betulinic acid and betulin, naturally occurring compounds found in the bark of trees, have been extensively studied for their role in inducing mitochondrial-dependent apoptosis in NSCLC cells. Betulinic acid has been shown to trigger apoptosis through mitochondrial dysfunction, oxidative stress, and caspase cascade activation [67,68]. Furthermore, betulin, derived from birch bark, also activates the intrinsic apoptotic pathway by increasing ROS production and modulating the Bax/Bcl-2 ratio [68,69,70]. Oleanolic acid induces apoptosis through both intrinsic and extrinsic pathways by activating caspase cascades. It also upregulates Bax and suppresses Survivin expression [71]. Ursolic acid, a compound found in various fruits, vegetables, and medicinal herbs, is a potent inducer of apoptosis, triggering mitochondrial dysfunction and the subsequent release of cytochrome c, which activates caspases in NSCLC cells [72,73,74]. Glycyrrhetinic acid, a component of licorice, promotes apoptosis through the MAPK/STAT3 pathway. Additionally, it induces apoptosis by modulating Bcl-2 expression and activating the caspase pathway [75,76]. Glycyrrhizin, derived from licorice root, exerts its apoptotic effects by regulating cyclooxygenase-2 (COX-2) and its downstream enzymes, including prostacyclin synthase (PGIS), prostaglandin E synthase (PGES) and thromboxane synthase (TxAS). Notably, glycyrrhizin effectively inhibits the expression of TxAS at both the transcriptional and translational levels [77]. Asiatic acid, a component of *Centella asiatica*, promotes apoptosis by inducing mitochondrial dysfunction and modulating the p53 and phosphatidylinositol-3-kinase (PI3K) signaling pathway [78,79].

### 3.2. Inhibition of Proliferation and Cell Growth

**Figure 4:**
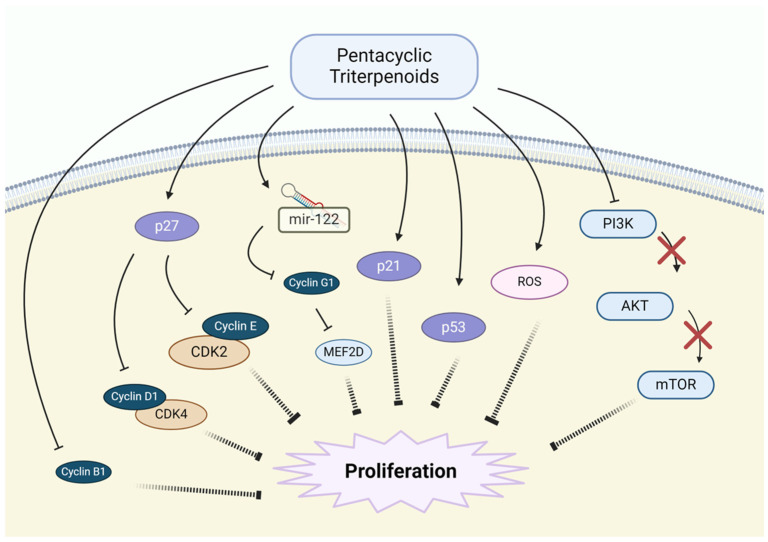
Inhibition of proliferation and cell growth by pentacyclic triterpenoids.

Pentacyclic triterpenoids have demonstrated the ability to inhibit the proliferation and growth of NSCLC cells through several key mechanisms. These compounds can interfere with the cell cycle, regulate key signaling pathways, and induce cellular stress, leading to a reduction in cancer cell growth (Figure 4).

Betulinic acid has been shown to inhibit cell proliferation in NSCLC by inducing cell cycle arrest at the G1/S phase. This occurs through the modulation of cyclin-dependent kinases (CDKs) and their regulatory cyclins, resulting in the downregulation of cell cycle progression. Furthermore, it has been found to activate the p53 pathway, which plays a crucial role in this process [68,80,81]. Betulin inhibits NSCLC cell proliferation by modulating key cell cycle regulators, including p27, p21, and cyclin-B1, -D, and -E. It also activates the AMPK pathway, which suppresses the proliferation of NSCLC cells [68,82]. Oleanolic acid exerts its inhibitory effect on NSCLC cell proliferation by inducing cell cycle arrest at both the G0/G1 and G2/M phases. This compound modulates the expression of key cell cycle regulators, such as cyclin D1, cyclin E, and CDK inhibitors, disrupting normal cell cycle progression. It also enhances reactive oxygen species (ROS) production, resulting in oxidative stress, which further inhibits cancer cell growth. Additionally, it modulates the mir-122/cyclin G1/MEF2D pathway, which is involved in regulating cell growth, contributing to the inhibition of NSCLC cell proliferation [83,84]. Ursolic acid inhibits NSCLC cell proliferation primarily by inducing G1 phase arrest. It downregulates the expression of cyclin-D1 and CDK4, key regulators of G1/S progression, thereby preventing the transition to the S phase. It also inhibits the AKT/mTOR signaling pathway, which is known to regulate cell growth and proliferation. By increasing ROS levels, it induces oxidative stress, which further limits cell proliferation [85,86,87]. Glycyrrhizin inhibits NSCLC cell proliferation by regulating the cell cycle. It has been shown to induce G0/G1 phase arrest by modulating the expression of cyclin-D1, -D3, and -E2, as well as CDKs 4, 6, and 2 while increasing the levels of p27, p21, p18, and p16, which are cyclin-dependent kinase inhibitors (CKIs) [88,89]. It also inhibits High Mobility Group Box 1 (HMGB1), a cytokine related to cancer progression, by suppressing the JAK/STAT signaling pathway [90]. Asiatic acid inhibits the proliferation of NSCLC cells by inducing cell cycle arrest at the G0/G1 phase. This occurs through the downregulation of cyclin-D1 and CDK2, leading to the inhibition of cell cycle progression. It also induces oxidative stress by elevating ROS levels, which triggers DNA damage and disrupts the proliferation of cancer cells. Additionally, asiatic acid regulates the PI3K/AKT signaling pathway, which plays a pivotal role in cancer cell growth and survival. By modulating this pathway, it effectively suppresses NSCLC cell proliferation [78,91].

### 3.3. Inhibition of Angiogenesis

**Figure 5:**
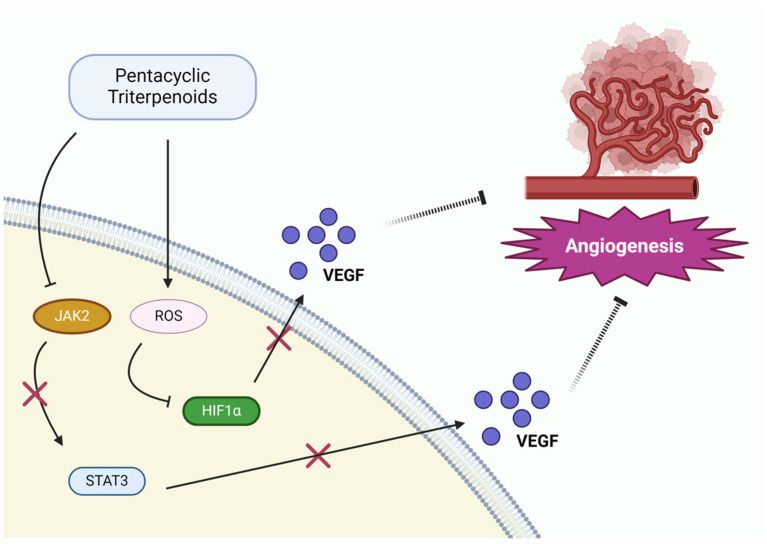
Inhibition of angiogenesis by pentacyclic triterpenoids.

Angiogenesis, the process of new blood vessel formation, is essential for the growth and metastasis of solid tumors, including NSCLC [106,107]. By promoting angiogenesis, tumors can secure the necessary oxygen and nutrients required for rapid expansion [108]. Pentacyclic triterpenoids, including ursolic acid and oleanolic acid, have been extensively studied for their anti-angiogenic effects in NSCLC, providing a promising approach to limit tumor growth and metastasis (Figure 5).

Oleanolic acid exerts its anti-angiogenic effects by inhibiting VEGF secretion and downregulating the PI3K/Akt signaling pathway, which is involved in endothelial cell proliferation and migration. It also interferes with endothelial cell function, preventing their migration and the formation of new blood vessel networks [71]. These actions help limit tumor angiogenesis and inhibit cancer spread. Similarly, ursolic acid inhibits angiogenesis by downregulating vascular endothelial growth factor (VEGF) expression, a key regulator of blood vessel formation. In addition, it suppresses the activation of hypoxia-inducible factor-1 alpha (HIF-1α), a transcription factor that increases VEGF production under low oxygen conditions. By blocking these pro-angiogenic signals, ursolic acid reduces the ability of tumors to develop new blood vessels, thereby restricting their growth potential [74,86,92].

### 3.4. Suppression of Metastasis

**Figure 6:**
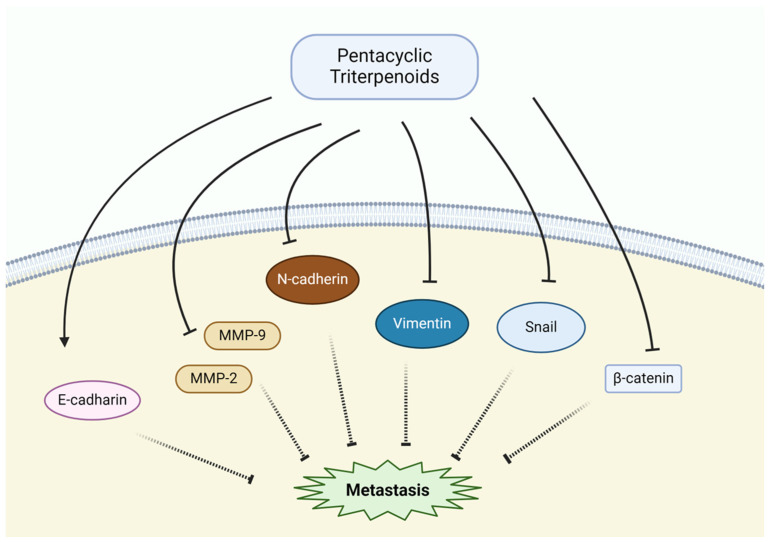
Anti-metastatic effects of pentacyclic triterpenoids.

Metastasis, the spread of cancer cells to distant organs, is a critical step in the progression of NSCLC and other malignancies. The inhibition of metastasis is an essential therapeutic strategy, as it can significantly improve survival rates and prevent the further spread of cancer. Several pentacyclic triterpenoids, including betulinic acid, betulin, ursolic acid, glycyrrhizin, and asiatic acid, have demonstrated potent anti-metastatic effects through various mechanisms, such as inhibiting cancer cell invasion, migration, epithelial-to-mesenchymal transition (EMT), and matrix metalloproteinase (MMP) activity (Figure 6).

Betulinic acid exerts its anti-metastatic effects by suppressing Skp2-mediated E-cadherin ubiquitination, thus inhibiting cancer cell invasion [93]. Additionally, it inhibits NSLC metastasis by blocking F-actin polymerization, which regulates the cytoskeletal movement involved in migration [67]. Betulin exerts anti-metastatic effects by targeting MMP-2 and MMP-9, which are essential for cancer cell invasion. It also modulates the Wnt/β-catenin signaling pathway, which regulates cancer cell proliferation and migration. By inhibiting this pathway, betulin suppresses cancer cell invasiveness and limits tumor metastasis [68,69]. Ursolic acid plays a significant role in inhibiting EMT, a critical process that enables cancer cells to become more invasive and migratory. This transition is driven by transcription factors such as Snail and Twist, which are effectively downregulated by ursolic acid. By suppressing EMT, ursolic acid reduces the motility and invasiveness of cancer cells. Furthermore, it decreases the expression of MMP-2 and MMP-9, enzymes responsible for degrading the extracellular matrix and facilitating tissue invasion, thereby limiting tumor metastasis [74,86,94]. Glycyrrhizin inhibits cancer cell invasion and migration through multiple mechanisms. It reduces the expression and activity of MMP-2 and MMP-9 and suppresses the NF-κB signaling pathway, which is critical for the regulation of genes involved in metastasis [95]. Asiatic acid suppresses metastasis by upregulating the expression of E-cadherin at both the mRNA and protein levels, while the expression levels of EMT markers such as Snail, N-cadherin, vimentin, and β-catenin are downregulated [96].

### 3.5. Modulation of Inflammatory Pathways

**Figure 7:**
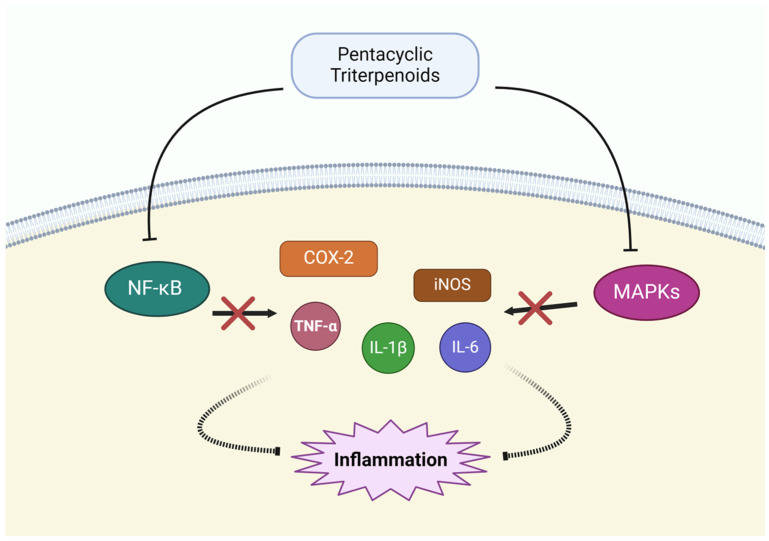
Modulation of inflammatory pathways by pentacyclic triterpenoids.

Chronic inflammation plays a significant role in the development and progression of cancer, including NSCLC [109,110,111,112]. It creates a tumor-promoting microenvironment by stimulating cell proliferation, invasion, and metastasis. Therefore, targeting inflammatory pathways is a promising strategy for cancer therapy. Several pentacyclic triterpenoids, such as oleanolic acid, ursolic acid, glycyrrhetinic acid, glycyrrhizin, and asiatic acid, have demonstrated potent anti-inflammatory effects by modulating key inflammatory signaling pathways, particularly targeting the NF-κB pathway, MAPK signaling, and pro-inflammatory cytokines (Figure 7).

Oleanolic acid exerts anti-inflammatory effects by inhibiting both NF-κB and MAPK signaling pathways. These pathways regulate inflammatory genes, including COX-2 and inducible nitric oxide synthase (iNOS), which promote tumor growth and progression. Reducing the activity of these pathways lowers the expression of pro-inflammatory markers and limits the inflammatory response in the TME [97]. Ursolic acid also targets NF-κB activation. NF-κB regulates the production of inflammatory mediators such as TNF-α, IL-1β, and IL-6, which are often upregulated in cancer. It downregulates these cytokines, thereby reducing the inflammation that drives tumor growth, invasion, and metastasis. Additionally, it inhibits the expression of COX-2 and iNOS, both of which are key enzymes involved in inflammation and cancer progression [98]. Glycyrrhizin and glycyrrhetinic acid are other pentacyclic triterpenoids with anti-inflammatory effects. It inhibits the NF-κB pathway, preventing the activation of genes involved in inflammation and tumor progression. It also reduces the expression of pro-inflammatory cytokines such as TNF-α, IL-1β, and IL-6. By modulating these inflammatory responses, glycyrrhizin creates a less favorable environment for tumor progression [99]. Asiatic acid, derived from *Centella asiatica*, modulates inflammatory pathways through NF-κB inhibition. NF-κB regulates pro-inflammatory cytokines such as TNF-α, IL-1β, and IL-6, which contribute to tumor progression and metastasis. By suppressing NF-κB activation, asiatic acid reduces the production of these cytokines, alleviating chronic inflammation that supports cancer development [100].

### 3.6. Immune Modulation

**Figure 8:**
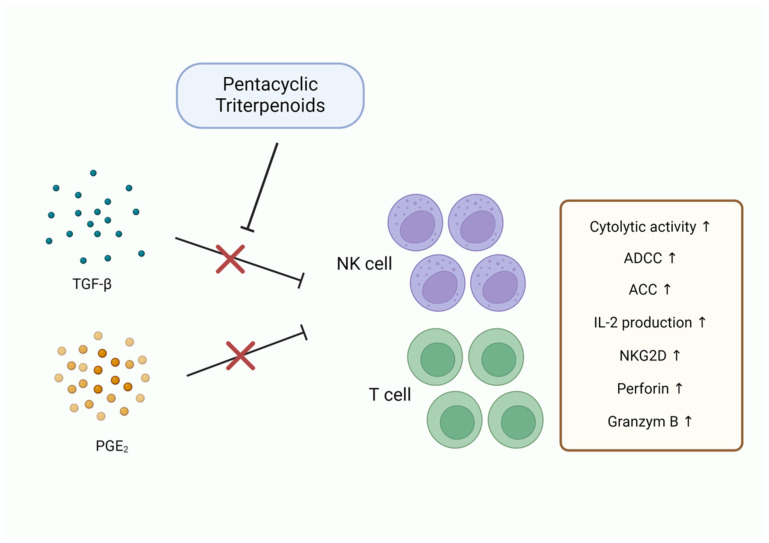
Immune modulation by pentacyclic triterpenoids.

Pentacyclic triterpenoids have demonstrated promising immunomodulatory effects by modulating various components of the immune system. These compounds affect the activity of immune cells, such as T cells, and natural killer (NK) cells, enhancing anti-cancer immune responses (Figure 8).

Betulinic acid and betulin have been shown to promote T cell activation and anti-tumor activity by increasing cytokines that support T cell proliferation and differentiation [101,102]. Furthermore, betulin enhances the cytotoxic activity of NK cells by counteracting tumor growth factor β1 (TGF-β1)- and prostaglandin E2 (PGE2)-induced immunosuppression in the TME, thereby promoting their ability to eliminate cancer cells [103]. Ursolic acid and glycyrrhizin increase NK cell activity through antibody-dependent, cell-mediated cytotoxicity (ADCC) and antibody-dependent, complement-mediated cytotoxicity (ACC). Additionally, these compounds upregulate the production of IL-2, which enhances cell-mediated immune response by stimulating immune cells including T cells and NK cells [104]. Asiatic acid modulates the TME by rebalancing TGF-β1/Smad signaling. By attenuating the inhibitory effect of TGF-β1 on NK cells, it promotes NK cell production through Id2- and IRF2-associated mechanisms [105].

## 4. Improving the Therapeutic Potential of Pentacyclic Triterpenoids in NSCLC Applications and Future Directions

Pentacyclic triterpenoids show significant promise as potential anticancer agents, but their therapeutic potential in NSCLC treatment is often hindered by challenges such as poor bioavailability, rapid metabolism, and insufficient tumor targeting. To improve their efficacy, strategies must be developed to overcome these obstacles and enhance tumor-specific delivery. Advanced drug delivery systems, such as nanoparticles, liposomes, and conjugates, are being explored to increase the solubility, stability, and targeted delivery of pentacyclic triterpenoids to lung tumors, thus maximizing their anticancer effects while minimizing systemic toxicity. Despite these limitations, pentacyclic triterpenoids exhibit diverse mechanisms of action, such as modulating TME, inhibiting angiogenesis, reducing inflammation, and regulating immune responses. The combination of these compounds with other agents like inhibitors of drug-metabolizing enzymes or efflux transporters may help to overcome resistance and enhance their therapeutic efficacy. Their integration into biomarker-driven treatment regimens could optimize therapeutic outcomes, especially in resistant or advanced stages of NSCLC.

### 4.1. Optimizing Drug Formulation and Delivery

A major challenge in the clinical application of pentacyclic triterpenoids is their poor bioavailability [113]. Many of these compounds have low water solubility, which limits their absorption and distribution within the body. To address this limitation, nanoformulations and liposomal delivery systems have been explored as potential solutions [113,114,115]. These approaches can enhance the solubility, stability, and targeted delivery of triterpenoids, ensuring higher drug concentrations at the tumor site while minimizing off-target effects. For example, lipid-based nanoparticles or polymeric micelles can encapsulate pentacyclic triterpenoids and protect them from rapid degradation, allowing for sustained release at the tumor location. Additionally, conjugation with targeting ligands, such as antibodies or small molecules, can improve selective uptake by cancer cells, further enhancing their therapeutic efficacy [116,117,118].

### 4.2. Combination Strategies to Overcome Chemoresistance

Another strategy for enhancing the efficacy of pentacyclic triterpenoids is their combination with other therapeutic modalities, including chemotherapy, radiotherapy, and immunotherapy. Despite advances in therapeutic regimens, the 5-year survival rate for lung cancer remains approximately 20–30%, largely due to late-stage diagnoses and chemoresistance. Conventional chemotherapy is still the standard treatment for lung cancer patients; however, overcoming obstacles like chemoresistance is critical to improving treatment outcomes.

Chemoresistance poses a significant barrier to successful NSCLC treatment, driven by a complex interplay of genetic, epigenetic, and microenvironmental factors [119,120]. One key mechanism involves the overexpression of ATP-binding cassette (ABC) transporters, such as P-glycoprotein (P-gp), which actively pump chemotherapeutic drugs out of cancer cells, reducing their intracellular concentrations and efficacy [121,122,123]. Survival signaling pathways, including PI3K/AKT/mTOR and NF-κB, further promote cell survival and proliferation despite chemotherapeutic stress. NSCLC cells also evade apoptosis through upregulated anti-apoptotic proteins like Bcl-2 and downregulated pro-apoptotic proteins such as Bax. Additionally, tolerance to oxidative stress enables cancer cells to neutralize chemotherapy-induced reactive oxygen species (ROS) by enhancing their antioxidant defenses. EMT further contributes to chemoresistance by increasing cell plasticity and promoting invasive phenotypes that are less responsive to conventional therapies.

Pentacyclic triterpenoids offer promising solutions to counteract these mechanisms of chemoresistance in NSCLC. These compounds directly inhibit the expression and activity of P-gp, leading to increased intracellular drug retention and improved efficacy of chemotherapeutic agents such as cisplatin and doxorubicin [124]. Moreover, pentacyclic triterpenoids suppress survival signaling pathways, including PI3K/AKT/mTOR and NF-κB, reducing cancer cell survival and enhancing chemotherapy sensitivity. They also restore apoptotic balance by upregulating pro-apoptotic proteins like Bax, suppressing anti-apoptotic proteins such as Bcl-2, and activating caspase-dependent programmed cell death. Furthermore, these compounds overwhelm the antioxidant defenses of cancer cells by inducing ROS generation, creating oxidative stress that sensitizes cancer cells to chemotherapeutic agents. Finally, pentacyclic triterpenoids reverse EMT by downregulating mesenchymal markers such as vimentin and upregulating epithelial markers like E-cadherin, restoring chemosensitivity and inhibiting metastatic potential.

By addressing the multifaceted mechanisms underlying chemoresistance, pentacyclic triterpenoids represent a powerful adjunct to existing therapeutic regimens.

### 4.3. Targeting the TME

In addition to their direct anti-cancer effects, pentacyclic triterpenoids can modulate the TME to improve therapeutic efficacy. These compounds regulate inflammation, reduce oxidative stress, and inhibit angiogenesis—processes critical for tumor growth and metastasis. By targeting tumor stroma, pentacyclic triterpenoids disrupt the supportive environment for cancer cells while enhancing the efficacy of other therapeutic agents.

Pentacyclic triterpenoids modulate inflammatory pathways by downregulating pro-inflammatory cytokines, such as IL-6 and TNF-α, thereby mitigating the chronic inflammation that drives tumor progression. Their antioxidant properties further reduce oxidative stress within the TME, disrupting cancer cell survival and sensitizing tumors to conventional therapies. Additionally, these compounds inhibit angiogenesis by suppressing VEGF signaling, which reduces the formation of new blood vessels that supply nutrients and oxygen to tumors. This anti-angiogenic effect effectively starves the tumor, limiting its growth and metastatic potential. By reprogramming the TME, pentacyclic triterpenoids not only suppress tumor growth and metastasis but also enhance the effectiveness of conventional therapies.

These properties make them valuable candidates for integration into multimodal cancer treatment strategies, potentially improving outcomes when used alongside standard therapies.

### 4.4. Personalized Medicine and Biomarker Development

Personalized medicine provides a promising pathway to enhance the efficacy of pentacyclic triterpenoids in lung cancer therapy. Identifying predictive biomarkers to determine which patients are likely to benefit from pentacyclic triterpenoid treatment is a critical step toward improving clinical outcomes. The molecular profiling of lung cancer, including the analysis of key genomic alterations, expression patterns, and immune signatures, will enable the identification of patient subsets that respond favorably to pentacyclic triterpenoid therapy. Such biomarkers could include mutations in oncogenes and tumor suppressors, levels of inflammation-related cytokines, or the expression of oxidative stress-related proteins that pentacyclic triterpenoids are known to target.

Advances in technologies such as next-generation sequencing (NGS), single-cell RNA sequencing, and proteomics provide deeper insights into tumor heterogeneity and the molecular mechanisms influenced by pentacyclic triterpenoids. These insights can guide the design of precision drug delivery systems to enhance tumor-specific uptake while minimizing off-target effects. Combining molecular profiling with the real-time monitoring of treatment responses through liquid biopsies can further refine therapeutic regimens and allow for dynamic adjustments during treatment.

The integration of personalized medicine with pentacyclic triterpenoid-based therapies also opens opportunities for synergistic combinations with targeted agents, immunotherapies, or chemotherapies. Tailoring these combinations to the unique molecular and immunological landscape of each patient’s tumor could significantly improve the therapeutic efficacy of pentacyclic triterpenoids in lung cancer. This approach has the potential to transition pentacyclic triterpenoids from promising preclinical agents to effective components of individualized lung cancer treatment protocols.

### 4.5. Clinical Trials and Translational Research

Although much of the research on pentacyclic triterpenoids has been conducted in vitro or in animal models, there is a growing push toward clinical trials to assess their safety and efficacy in human lung cancer patients. The pharmacokinetics and bioavailability of pentacyclic triterpenoids need to be optimized to improve their clinical application. Strategies such as nanoparticle-based delivery systems, liposomal formulations, and prodrug approaches are being explored to enhance their stability, solubility, and targeted delivery to tumor sites.

Given their relatively low toxicity and broad spectrum of action, these compounds have the potential to be developed into clinically relevant agents, either as part of combination therapies or as stand-alone treatments. Furthermore, their ability to modulate the TME, enhance immune responses, and overcome drug resistance mechanisms makes them attractive candidates for integration into multimodal treatment regimens. Continued advancements in preclinical research, coupled with the establishment of well-designed clinical trials, are essential to fully realize the therapeutic potential of pentacyclic triterpenoids in lung cancer. Success in these endeavors could pave the way for new, more effective treatments, offering hope for improved outcomes in lung cancer patients.

### 4.6. Future Directions

Future research on pentacyclic triterpenoids should prioritize overcoming chemoresistance, a critical challenge in lung cancer treatment. These compounds have shown potential in modulating key pathways such as PI3K/AKT/mTOR, NF-κB, and Wnt/β-catenin, enhancing apoptosis, inhibiting P-glycoprotein activity, and reprogramming the TME. Additionally, their ability to reverse EMT highlights their role in restoring chemosensitivity and inhibiting metastasis.

Advancing drug delivery systems, such as nanoparticles and liposomes, is essential to improve tumor-specific targeting and sustained release. These systems maximize therapeutic efficacy while minimizing systemic toxicity. Combining pentacyclic triterpenoids with conventional therapies, such as chemotherapy and immunotherapy, holds promise for overcoming resistance and enhancing treatment outcomes.

Furthermore, leveraging molecular profiling and advanced technologies like single-cell sequencing can identify predictive biomarkers for chemoresistance. Such biomarkers could guide personalized medicine approaches, enabling tailored pentacyclic triterpenoid-based therapies that match individual tumor profiles. This strategy could significantly improve patient selection and optimize clinical outcomes.

Most studies on pentacyclic triterpenoids have focused on NSCLC, but the exploration of their potential in SCLC is limited. SCLC is characterized by frequent alterations in key molecular pathways, including the dysregulation of the MYC family of oncogenes [125,126,127], the inactivation of tumor suppressors such as TP53 and retinoblastoma (RB)1 [127,128,129], and the aberrant signaling of the Notch, Hedgehog, and PI3K/AKT/mTOR pathways [130,131,132,133]. In addition, overexpression of DNA damage response proteins and dependence on anti-apoptotic proteins such as BCL-2 contribute to its aggressive nature and therapeutic resistance [134]. Pentacyclic triterpenoids, which can modulate apoptosis through BCL-2 inhibition and modulate the PI3K/AKT/mTOR signaling pathway, may be promising candidates to target these vulnerabilities.

With continued progress in delivery technologies, mechanistic insights, and clinical trial designs, pentacyclic triterpenoids hold the potential to become integral components of future lung cancer treatment regimens. Their ability to address chemoresistance and improve the efficacy of standard therapies offers hope for better outcomes in resistant and advanced stages of lung cancer.

## 5. Conclusions

Pentacyclic triterpenoids have emerged as promising candidates in lung cancer therapy, offering diverse mechanisms such as apoptosis induction, the suppression of proliferation and metastasis, and the modulation of TME. These compounds hold the potential to complement conventional therapies, addressing challenges like chemoresistance and enhancing immune responses. When compared to existing or emerging lung cancer therapies, pentacyclic triterpenoids offer distinct advantages in targeting multiple pathways involved in tumor progression. While traditional treatments like chemotherapy, radiotherapy, and targeted therapies often have limitations such as drug resistance, toxicity, and side effects, pentacyclic triterpenoids exhibit lower toxicity and a broader spectrum of action, making them suitable for combination therapies. For instance, the ability of these compounds to modulate TME and enhance immune responses offers a complementary approach to current immune checkpoint inhibitors and other immunotherapies. However, clinical translation faces hurdles, including poor bioavailability and pharmacokinetics, requiring advancements in delivery systems and biomarker-guided approaches. Comparing pentacyclic triterpenoids with emerging therapies, such as small molecule inhibitors, monoclonal antibodies, and immune-based treatments, emphasizes the need for improving the pharmacokinetic properties of these compounds to increase their clinical utility. Additionally, identifying biomarkers that can predict the efficacy of pentacyclic triterpenoids in specific lung cancer subtypes may provide a more targeted approach to their application. Continued preclinical and clinical research will be vital to unlock their therapeutic potential, paving the way for more personalized and effective lung cancer treatments.

## Figures and Tables

**Table 1:** Summary of molecular mechanisms of pentacyclic triterpenoids in NSCLC. (“↑” means upregulation and “↓” means downregulation).

Mechanism	Pentacyclic Triterpenoids	Target Pathway	References
Induction of Apoptosis	Lupeol	Bax↑, Bcl-2↓	[63,64,65,66]
	Betulinic acid	ROS↑, Caspase activation↑	[67,68]
	Betulin	ROS↑, Bax/Bcl-2 ratio	[68,69,70]
	Oleanolic acid	Caspase activation↑, Bax↑, Survivin↓	[71]
	Ursolic acid	Release of cytochrome C, Caspase activation↑	[72,73,74]
	Glycyrrhetinic acid	MAPK/STAT3 pathway↓, Bcl-2↓	[75,76]
	Glycyrrhizin	TxAS↓	[77]
	Asiatic acid	p53↑, PI3K pathway↓	[78,79]
Inhibition of Proliferation	Betulinic acid	CDKs↓, p53↑	[68,80,81]
	Betulin	p27,p21↑, Cyclin-B1,-D,-E↓, AMPK pathway↑	[68,82]
	Oleanolic acid	CDK inhibitors↑, Cyclin-D1↓, ROS↑	[83,84]
	Ursolic acid	Cyclin-D1↓, CDK4↓, AKT/mTOR pathway↓	[85,86,87]
	Glycyrrhizin	p27, p21, p18, p16↑ Cyclin-D1,-D3,E2↓, CDK4,6,2↓, HGMB1↓	[88,89,90]
	Asiatic acid	Cyclin-D1, CDK2↓, ROS↑, PI3K/AKT pathway↓	[78,91]
Inhibition of Angiogenesis	Oleanolic acid	VEGF↓, PI3K/AKT pathway↓	[71]
	Ursolic acid	VEGF↓, HIF-1α↓	[74,86,92]
Suppression of Metastasis	Betulinic acid	E-cadherin ubiquitination↓, F-actin polymerization↓	[67,93]
	Betulin	MMP-2,-9↓, Wnt/β-catenin↓	[68,69]
	Ursolic acid	MMP-2,-9↓	[74,86,94]
	Glycyrrhizin	MMP-2,-9↓, NF-κB pathway↓	[95]
	Asiatic acid	E-cadherin↑, Snail↓, N-cadherin↓, Vimentin↓, β-catenin↓	[96]
Modulation of Inflammatory Pathway	Oleanolic acid	NK-κB pathway↓, MAPK pathway↓	[97]
	Ursolic acid	TNF-α↓, IL-1β↓, IL-6↓, COX-2↓, iNOS↓	[98]
	Glycyrrhizin, Glycyrrhetinic acid	NK-κB pathway↓, TNF-α↓, IL-1β↓, IL-6↓	[99]
	Asiatic acid	NK-κB pathway↓, TNF-α↓, IL-1β↓, IL-6↓	[100]
Immune Modulation	Betulinic acid	T cell activation	[101,102]
	Betulin	NK cell activation, TGF-β1↓, PGE_2_↓	[103]
	Ursolic acid	ADCC↑, ACC↑, IL-2↑, NK cell activation	[104]
	Asiatic acid	TGF-β1/Smad signaling↓, NK cell activation	[105]

## Data Availability

Not applicable.
